# Elevated Plasma D-Dimer Levels Correlate with Long Term Survival of Gastric Cancer Patients

**DOI:** 10.1371/journal.pone.0090547

**Published:** 2014-03-11

**Authors:** Long Liu, Xi Zhang, Bing Yan, Qunhao Gu, Xiaodong Zhang, Jianpeng Jiao, Dazhi Sun, Ning Wang, Xiaoqiang Yue

**Affiliations:** 1 Department of Traditional Chinese Medicine, Changhai Hospital, Second Military Medical University, Shanghai, China; 2 Department of Anatomy, Second Military Medical University, Second Military Medical University, Shanghai, China; 3 Department of Traditional Chinese Medicine, Changzheng Hospital, Second Military Medical University, Shanghai, China; 4 Department of General Surgery, Yueyang Hospital, Shanghai University of Traditional Chinese Medicine, Shanghai, China; 5 Department of Oncology, Changhai Hospital, Second Military Medical University, Shanghai, China; Ottawa Hospital Research Institute, Canada

## Abstract

**Background:**

Increasing evidence indicated plasma D-dimer could be regarded as a marker in cancers, however, its role in gastric cancer is still largely unknown.

**Methods:**

Plasma D-dimer levels were measured by enzyme linked fluorescent immunoassays and evaluated by receiver operating characteristic (ROC) curves for peritoneal dissemination in gastric cancer and healthy subjects. The overall survival (OS) characteristics were determined using Kaplan–Meier and Cox regression analyses.

**Results:**

The average of the plasma D-dimer levels for gastric cancer patients was significantly higher than the healthy subjects. A Spearman correlation analysis showed that plasma D-dimer levels correlated with the depth of invasion, lymph node metastasis, peritoneal dissemination, distant metastasis, tumor size and TNM stage. The mean plasma D-dimer level was 2.20±1.51 µg/mL in peritoneal dissemination patients and 1.01±0.79 µg/mL in non-peritoneal dissemination patients (P<0.001). Additionally, the mean plasma D-dimer concentration in patients alive at the final follow-up evaluation was 0.79±0.72 µg/mL,which was significantly lower than the amounts determined for the deceased patients (1.36±1.13 µg/mL) (P<0.001). The AUC of D-dimer was 0.833 (95%CI: 0.780–0.885). At a cut-off value of 1.465 µg/mL, the D-dimer measurement had a sensitivity of 78.00%, a specificity of 83.76% and an accuracy of 82.59%. The median OS was 48.10 months (95% CI: 43.88–52.31) in patients with plasma D-dimer levels less than 1.465 µg/mL and 22.39 months (95% CI: 16.95–27.82) in patients with plasma D-dimer levels exceeding 1.465 µg/mL (log-rank test, P<0.001). Importantly, plasma D-dimer levels exceeding 1.465 µg/mL were significantly associated with poor OS, as determined using a multivariate Cox regression analysis (hazard ratio [HR], 2.28; 95%CI: 1.36–3.81; P = 0.002).

**Conclusions:**

Plasma D-dimer levels are increased in gastric cancer patients and may be a valuable biomarker for peritoneal dissemination, with high D-dimer levels predicting poor outcomes for gastric cancer patients.

## Introduction

Gastric cancer is the fourth most prevalent malignant cancer worldwide and is the second most frequent cause of cancer death [1]. Almost two thirds of the cases occur in developing countries with 42% of the cases in China [2]. Epidemiological studies have shown that in recent years, in addition to the well-known risk factors like genetic mutation, dietary, tobacco and *Helicobacter pylori* (HP) infection, the consequent atrophic gastritis have also been regarded as a primary risk factor for gastric cancer, which was previously thought as precancerous lesions. In recent years, the relationship between atrophic gastritis and gastric cancer has been further confirmed by a group of retrospective studies [3]–[4].

Commonly diagnosed at a very late stage, approximately one-half of the gastric cancer patients present with unresectable disease and nearly one-third of the patients exhibit peritoneal dissemination [5]. As peritoneal dissemination adversely impacts gastric cancer survival rates, the 5-year survival rate for advanced or metastatic gastric cancer is only 5–20% [6]–[7]. Difficult to diagnose prior to a laparoscopy, peritoneal metastasis is a crucial factor for the prognosis in gastric cancer. Computed tomography (CT) and diagnostic laparoscopy can both increase the rate of definitive diagnoses, with the diagnostic value of these techniques limited by cost, risks, and inconvenience. Given these limitations, the development of noninvasive, sensitive and specific biomarkers that enable the detection of peritoneal dissemination would be beneficial.

Coagulation abnormalities have been observed in patients with malignant tumors [8]–[10] for approximately one-half of the patients with malignant disease. Over 90% of the patients with metastatic lesions manifested with abnormalities in clotting and/or fibrinolysis, including antithrombin-III (AT-III) complexes, fibrinopeptides A (FPA) and D-dimer [11]. An increased rate of thrombosis was first documented in patients with gastrointestinal cancer in the 1960s. A recent study of fifty-two gastric cancer patients demonstrated increased levels of thrombin-antithrombin (TAT) complex and thrombin activatable fibrinolysis inhibitor (TAFI) [12], with increased levels of D-dimer in stageIV gastric cancer patients. In a study of 1178 patients over a two-year period, Ay et al. found that in a subgroup of 50 gastric cancer patients, increased D-dimer plasma levels were associated with reduced survival and were a significant risk factor for mortality [13]. In a study of 110 patients with adenocarcinoma of the stomach. Kwon et al. determined that D-dimer levels correlated with the depth of invasion, clinical stage and lymph node involvement in patients with operable gastric cancer [14].

**Figure 1:**
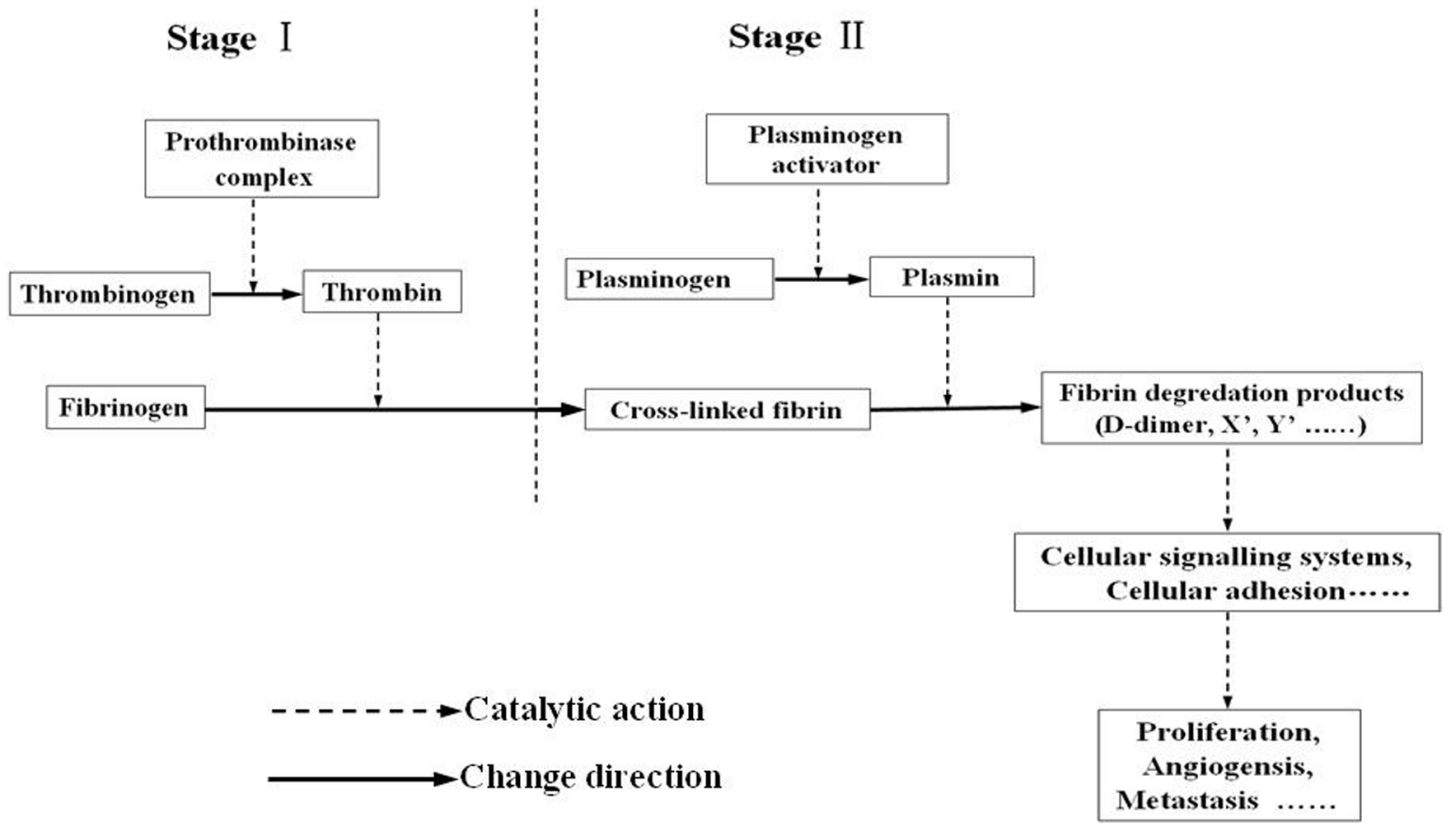
Pathophysiology of D-dimer. Stage I was the process of blood coagulation, Stage II was the process of fibrinolysis, as the degradation product of fibrin, D-dimer can promote the growth and metastasis of tumors.

As a degradation product of fibrin, D-dimer was produced when cross-linked fibrin was degraded by plasmin-induced fibrinolytic activity (Fig. 1). Researchers recently reported that D-dimer can not only affect cellular signaling systems, promote cell proliferation and induce angiogensis [15], but also stimulate the cellular adhesion of tumor cells to endothelial cells, affect platelets and extra-cellular matrix (ECM), and ultimately, induce the growth and spread of tumors [16] (Fig. 1).

These findings suggest that an examination of the plasma D-dimer levels could be a useful predicting marker for peritoneal dissemination in gastric cancer. To the best of our knowledge, a long term study on the association between plasma D-dimer levels and gastric cancer patient survival has not been reported. Additionally, the diagnostic performance of plasma D-dimer levels for peritoneal dissemination in gastric cancer has also not been studied. In this current study, plasma D-dimer levels in patients with gastric cancer and healthy controls were determined, and the diagnostic performance of an enzyme-linked fluorescent immunoassay for D-dimer levels was examined for determining peritoneal dissemination. Plasma D-dimer levels were also correlated with long time survival rates of gastric cancer patients.

## Materials and Methods

### 1.1: Patients

247 patients with pathologically proven gastric cancer receiving treatment at Changzheng Hospital, the Second Military Medical University, Shanghai, China between January 2002 and January 2004 were recruited for this study. 168 patients underwent radical gastrectomy, 46 patients underwent palliative gastrectomy and 23 patients treated with exploratory laparotomy, after surgery 50 patients with peritoneal dissemination were pathologically verified.

The disease progression in the GC patients was classified using the guidelines outlined in the seventh edition of the American Joint Committee [17]. Eligibility of the gastric cancer patients for inclusion within this study included the following requirements: 1) the patients were at least 18 years of age with pathologically proven gastric cancer; and 2) the patients had not received prior palliative therapy (including palliative chemotherapy and radiotherapy). Previous adjuvant (neo-adjuvant) chemotherapy was allowed if more than 6 months had elapsed between the end of the adjuvant (neo-adjuvant) therapy and the first relapse. Exclusion criteria from the study included the following conditions: 1) the patient was pregnant, lactating, or fertile without assuring adequate contraceptive measures; 2) the patient had previous malignant diagnoses, concurrent malignancies, or secondary tumors; 3) the patient had a history of thromboembolism, familial coagulopathy, active infections, or active disseminated intravascular coagulation; and 4) the patient had received either anticoagulant and anti-aggregate therapies. In the control group, 220 age- and sex-matched healthy volunteers (disease-free) were enrolled during the same time period. A summary of the data from the two groups is provided in Table 1.

**Table 1:** Demographic and baseline characteristics of the two patient groups.

	Mean ± SD or No. (%)	Mean ± SD or No. (%)
Variable	Gastric cancer (n = 247)	Control group (n = 220)
Age, years		
Mean(SD)	58.47±12.01	54.23±8.38
Gender, n(%)		
Male/Female	165(66.80)/82(33.20)	144(65.45)/76(34.55)
Pathological diagnosis, n(%)		
Adenocarcinoma	208(84.21)	
Signet-ring cell carcinoma	18(7.29)	
Others	21(8.50)	
Tumor size, n(%)		
<5 cm	178(72.06)	
≥5 cm	69(27.94)	
Invasion depth, n(%)		
T1	22(8.90)	
T2	37(14.98)	
T3	54(21.87)	
T4	134(54.254)	
Lymph node metastasis, n(%)		
N0	79(31.98)	
N1	33(13.36)	
N2	45(18.22)	
N3a	58(23.48)	
N3b	32(12.96)	
Distant metastasis, n(%)		
M0	178(72.06)	
M1	69(27.94)	
Peritoneal dissemination, n(%)		
Negative	197(79.76)	
Positive	50(20.24)	
TNM stage, n(%)		
I	44(17.81)	
II	57(23.08)	
III	77(31.17)	
IV	69(27.94)	
Survival, n(%)		
Yes	47(19.03)	
No	200(80.97)	

To evaluate the predictive value of D-dimer levels in the retrospective study described above, data from an additional cohort (n = 47) of patients with GC from January 2011 to July 2011 at the same hospital was prospectively collected and analyzed.

The study protocol was approved by the Chinese Ethics Committee of Human Resources at the Second Military Medical University. Written informed consent was obtained from the patients and the healthy controls.

### 1.2: Follow-up

Patient follow-up data were obtained through reviews of the hospital records, contact with family members of the patients, or reviews of the Cancer Registry of Shanghai. Patients were observed until December 31, 2010. Overall survival was defined as the interval between the dates of surgery and either the time of the last follow-up or death due to gastric cancer. Censoring occurred for patients still alive or deceased as a result of other reasons at the last follow-up.

### 1.3:Enzyme-linked fluorescent immunoassays for D-dimer levels

Venous blood samples were collected 2 days prior to surgery for the gastric cancer patients and at the physical examination day for the healthy volunteers. D-dimer levels were measured using an enzyme-linked fluorescent immunoassay method with a mini-Vidas device (BioMerieux SA). D-dimer levels <0.5 µg/mL were considered normal.

### 1.4: Statistical analyses

All statistical analyses were conducted using SPSS software (version 18.0 for Windows, SPSS Inc., Chicago, IL). All quantitative variables were expressed as the mean ± standard deviation (SD), unless otherwise stated. Categorical variables were expressed as the number (percentage). All categorical variables were analyzed using either the χ2 test or the Fisher exact test, as appropriate. The difference between the gastric cancer patients and the healthy controls was analyzed with a t-test. Correlations between the parameters were assessed according to the Spearman nonparametric test. The predictive performance of D-dimer levels for peritoneal dissemination was evaluated using a receiver operating characteristic (ROC) curves analysis. The sensitivities, specificities, positive predictive values (PPV), negative predictive values (NPV) and accuracies were calculated using a cut-off value that was selected from the ROC curve. The survival curves were calculated using the Kaplan–Meier method, and P-values were determined by the log-rank test for censored survival data. Both univariate and multivariate survival analyses were performed using the Cox proportional hazard model. For all tests, a 2-sided *P*-value of <0.05 was considered to be statistically significant.

## Results

### 2.1: Plasma D-dimer levels were significantly elevated in gastric cancer patients

**Figure 2:**
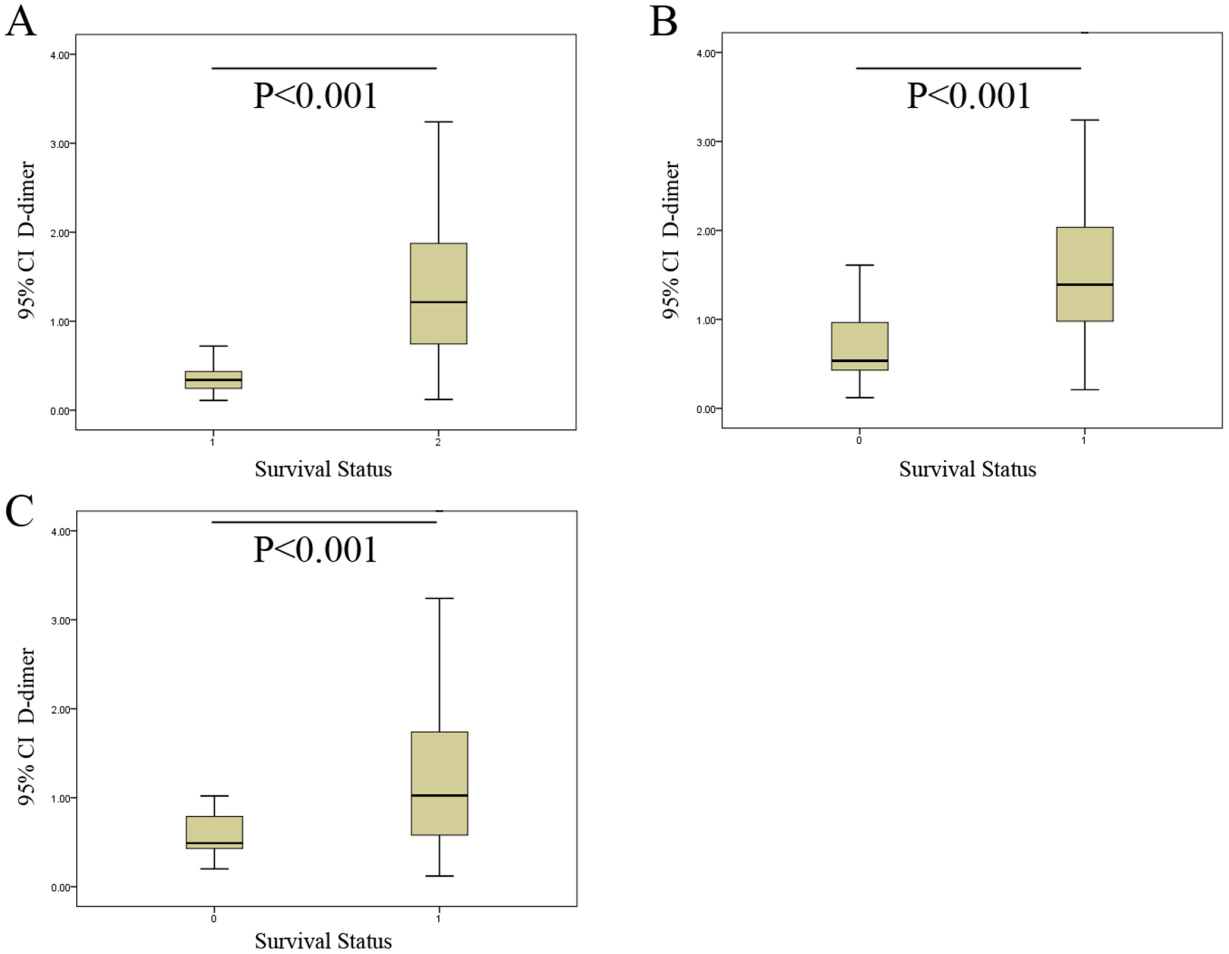
The differences of D-dimer levels. (A) Plasma levels of D-dimer in patients with gastric cancer (1.25±1.08 µg/mL) were significantly higher than the values determined for control subjects (0.37±0.20 µg/mL) (P<0.001). (B) The mean plasma D-dimer level of patients with peritoneal dissemination was 2.20±1.51 µg/mL, a value that was significantly higher than the measured amount for patients without peritoneal dissemination (1.01±0.79 µg/mL) (P<0.001). (C) The mean plasma D-dimer level in surviving patients was 0.79±0.720 µg/mL, a value that was significantly lower than the amount determined for the deceased patients (1.36±1.13 µg/mL) (P<0.001).

To determine the association of gastric cancer with elevated levels of D-dimer in the plasma, we measured the plasma levels of D-dimer in both patients with gastric cancer and healthy subjects using commercially available enzyme-linked fluorescent immunoassays. The average plasma D-dimer level of the gastric cancer patients (1.25±1.08 µg/mL) was significantly higher than the value determined for the control subjects (0.37±0.20 µg/mL) (P<0.001) (Fig. 2A). The mean plasma D-dimer level of patients with peritoneal dissemination was 2.20±1.51 µg/mL, significantly higher than the value determined for patients without peritoneal dissemination (1.01±0.79 µg/mL) (P<0.001) (Fig. 2B). In addition, the mean plasma D-dimer concentration in patients alive at the final follow-up examination was 0.79±0.720 µg/mL, significantly lower than the value determined for the patients who had died (1.36±1.13 µg/mL) (P<0.001) (Fig. 2C). A Spearman correlation analysis further showed that the plasma D-dimer levels correlated with invasion depth, lymph node metastasis, peritoneal dissemination and distant metastasis, tumor TNM stage, and tumor size (Table 2).

**Table 2:** Correlation between the plasma D-dimer levels and the clinicopathologic factors of gastric cancer patients.

Variables	Spearman correlation coefficient	P
Invasion depth	0.513	<0.001
Lymph node metastasis	0.389	<0.001
Distant metastasis	0.290	<0.001
Peritoneal dissemination	0.440	<0.001
TNM stage	0.489	<0.001
Tumor size	0.200	0.002

### 2.2: Diagnostic performance of D-dimer levels for peritoneal dissemination in gastric cancer patients

**Figure 3:**
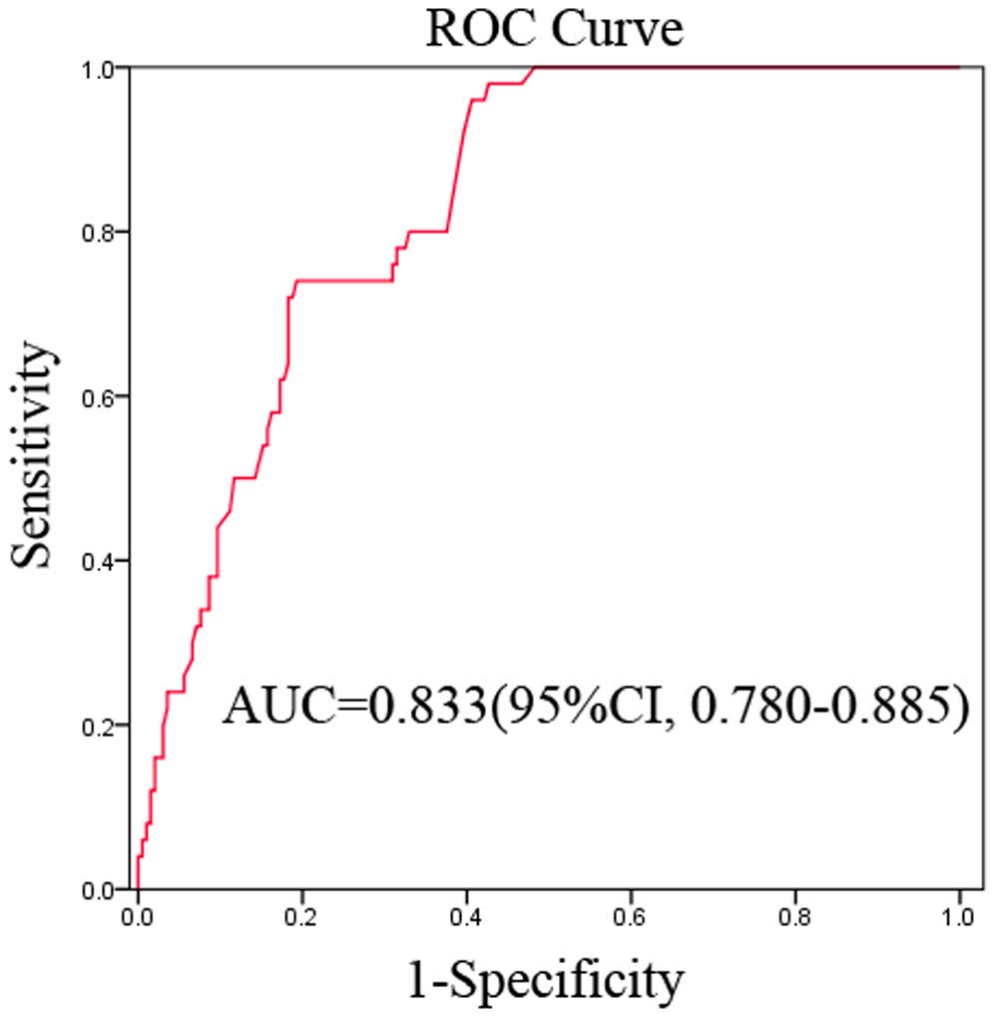
ROC curve analysis for the prediction of peritoneal dissemination. The Area under the ROC curve (AUC) indicates the diagnostic power of D-dimer levels (AUC = 0.833).

With the D-dimer levels correlating with peritoneal dissemination, the diagnostic performance of D-dimer for peritoneal dissemination in patients with gastric cancer was further investigated. We found that the AUC of the D-dimer was 0.833 (95% CI: 0.780–0.885) (Fig. 3).

The optimal cut-off value for the D-dimer concentration (1.465 µg/mL) was selected based on an ROC curve analysis. As shown in Table 3, the amount of D-dimer was determined to be an effective diagnostic marker for peritoneal dissemination. At a cut-off value of 1.465 µg/mL, the D-dimer concentration had a sensitivity of 78.00%, a specificity of 83.76% with a PPV of 54.93% and a NPV of 93.75%. The concentration of D-dimer also had an accuracy of 82.59%.

**Table 3:** The diagnostic power of D-dimer in differentiating peritoneal dissemination from the non-peritoneal dissemination in the Prospective Training Group.

		Actual status (No. of subjects)						
Cutoff value	Test	PD+	PD−	Sensitivity,%	Specificity,%	PPV,%	NPV,%	Accuracy,%
D-dimer, 1.465 µg/ml	PD+	39	32	78.00	83.76	54.93	93.75	82.59
	PD−	11	165					

Abbreviations: PD, peritoneal dissemination; +, positive; −, negative; PPV, positive predictive value; NPV, negative predictive value;.

In the prospective group, the sensitivity, specificity and accuracy were 75.00%, 85.71% and 82.98%, respectively (Table 4). The accuracy in the retrospective group was similar to that in the prospective group, suggesting that the D-dimer levels may be a valuable biomarker for the predicting of gastric cancer with peritoneal dissemination.

**Table 4:** The diagnostic power of D-dimer in differentiating peritoneal dissemination from the non-peritoneal dissemination in the Retrospective Verification Group.

		Actual statusNo. of subjects						
Cutoff value	Test	PD+	PD−	Sensitivity,%	Specificity,%	PPV,%	NPV,%	Accuracy,%
D-dimer1.465 µg/ml	PD+	9	5	75.00	85.71	64.29	90.91	82.98
	PD−	3	30					

Abbreviations: PD, peritoneal dissemination; +, positive; −, negative; PPV, positive predictive value; NPV, negative predictive value.

### 2.3: Plasma D-dimer levels and overall survival (OS)

As indicated by a univariate analysis, the depth of invasion, lymph node metastasis, peritoneal dissemination, distant metastasis, TNM stage, tumor size and the plasma D-dimer level significantly impacted the OS (Table 5). A multivariate survival analysis using the Cox proportional hazards model showed that the depth of invasion, lymph node metastasis, peritoneal dissemination, tumor size and the plasma D-dimer levels were independent risk factors for survival (Table 6).

**Table 5:** Univariate analysis of the prognostic factors for gastric cancer patients using the Cox regression model.

Parameters	Univariate analysis		
	Hazard ratio	95%CI	P value
Age, year(≥60/<60)	1.151	1.03–1.58	0.477
Gender (female/male)	1.028	0.76–1.38	0.855
Invasion depth (T1/T2/T3/T4)	1.99	1.68–2.36	<0.001
Lymph node metastasis (positive/negative)	3.08	2.22–4.28	<0.001
Distant metastasis (positive/negative)	6.42	4.59–8.97	<0.001
Peritoneal dissemination (positive/negative)	6.95	4.74–10.12	<0.001
TNM stage (I/II/III/IV)	2.42	2.05–2.87	<0.001
Tumor size, cm (≥5/<5)	2.21	1.66–2.94	<0.001
Pathological diagnosis (Adenocarcinoma/Signet-ring cell carcinoma/others)	1.14	0.91–1.42	0.27
D-dimer, µg/ml (≥1.465/<1.465)	2.52	1.87–3.89	<0.001

**Table 6:** Multivariate analysis of the prognostic factors for gastric cancer patients using the Cox regression model.

Parameters	Multivariate analysis		
	Hazard ratio	95%CI	P value
Invasion depth (T1/T2/T3/T4)	1.50	1.23–1.82	<0.001
Lymph node metastasis (positive/negative)	1.74	1.22–2.49	0.002
Peritoneal dissemination (positive/negative)	3.86	2.60–5.72	<0.001
Tumor size, cm (≥5/<5)	1.44	1.07–1.94	0.015
D-dimer, µg/ml (≥1.465/<1.465)	2.28	1.36–3.81	0.002

**Figure 4:**
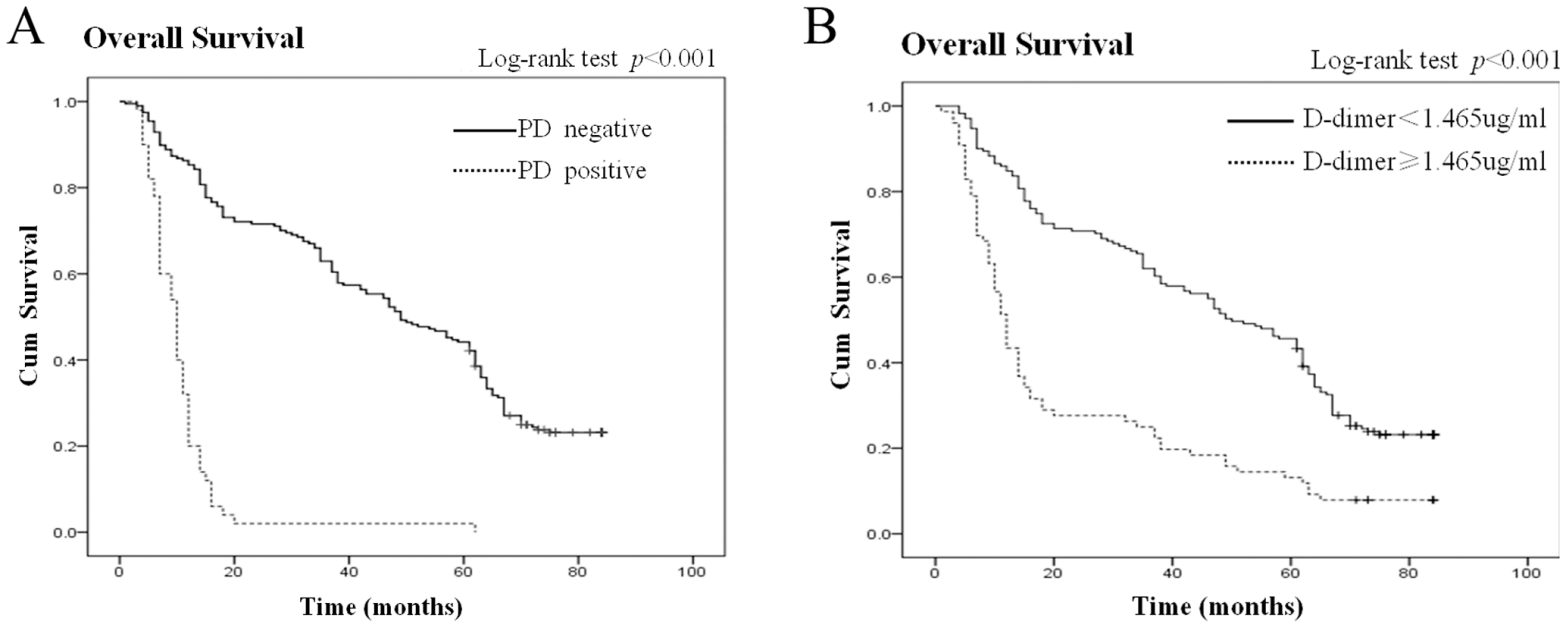
Plasma D-dimer and OS. (A) Kaplan-Meier curve for OS for gastric cancer patients stratified by peritoneal dissemination. Log-rank test, P<0.001 vs patients without peritoneal dissemination. (B) Kaplan-Meier curve for OS for gastric cancer patients stratified by plasma D-dimer levels (<1.465 µg/ml). Log-rank test, P<0.001 vs patients with plasma D-dimer levels ≥1.465 µg/ml.

The median duration of follow-up for the gastric cancer patients was 37.0 months (range, 1 to 84). An analysis of the OS of the gastric cancer patients stratified by peritoneal dissemination indicated that the median OS was 47.82 months (95% CI: 43.89–51.75) in patients without peritoneal dissemination and 10.64 months (95% CI: 8.31–12.97) in patients with peritoneal dissemination (Fig. 4A). The difference in OS between patients with high and low plasma D-dimer levels was statistically significant (log-rank test, P<0.001) with an HR of 3.86 (95% CI: 2.60–5.72). An analysis of the OS of the gastric cancer patients stratified by plasma D-dimer levels suggested that the median OS was 48.02 months (95% CI: 43.78–52.25) in patients with low plasma D-dimer levels (<1.465 µg/mL) and 22.92 months (95% CI: 17.46–28.38) in patients with high plasma D-dimer levels (≥1.465 µg/mL) (Fig. 4B). The difference in OS between the patients with high and low plasma D-dimer levels was statistically significant (log-rank test, P<0.001) with an HR of 2.28 (95% CI: 1.36–3.81).

## Discussion

The association between cancer and the activation of blood coagulation was first described more than 100 years ago. Commonly observed in cancer patients, the hypercoagulable state appears to act as an important risk factor for thrombosis complications and may play a role in disease progression. Among the degradation products resulting from the proteolytic actions of plasmin on fibrin, D-dimer is the smallest by-product, exhibiting unique characteristics. In a comparison of gastric ulcer patients and healthy controls, gastric cancer patients have increased levels of plasma D-dimer [18]. Consistent with the literature observations, this present study also found significantly higher D-dimer levels in the gastric cancer patients than the healthy subjects.

Recent studies have shown that an activation of the hemostatic system plays a role in tumor development, dissemination and metastasis. Plasminogen activators produce plasmin (an active serine protease), which has been implicated in both tumor invasion and the penetration of tumor cells into the circulatory system [19]–[20]. In previous studies, D-dimer levels were significantly increased in metastatic diseases [21]–[23]. In gastric cancer, these levels correlated with the depth of invasion, lymph node involvement and clinical stage [24]. In this study, D-dimer levels were significantly elevated in patients with peritoneal dissemination compared with those patients without peritoneal dissemination. These results are consistent with findings from a smaller study by Yumuck et al., demonstrating that D-dimer levels are significantly correlated with the presence of metastasis in gastric cancer patients [25].

High levels of plasma D-dimer have been found to be associated with poor prognosis in several malignant diseases, including lung cancer, colorectal cancer, ovarian cancer, gastric cancer and breast cancer [19], [26]–[29]. To the best of our knowledge, previous studies have not reported on the association between plasma D-dimer levels and long term survival of gastric cancer patients. In our study of 247 treatment-naive gastric cancer patients, low plasma D-dimer levels (<1.465 µg/ml) were associated with a significantly longer OS of 48.10 months (95% CI: 43.88–52.31) than the OS of 22.39 months for gastric cancer patients with high plasma D-dimer levels (≥1.465 µg/ml) (95% CI: 16.95–27.82). A multivariate analysis using the Cox regression model further indicated that D-dimer levels were an independent prognostic factor for survival in gastric cancer patients. Those gastric cancer patients with D-dimer levels ≥1.465 µg/mL were 2.28-fold more likely to die than those patients with D-dimer levels below this cut-off value (<1.465 µg/mL).

In previous studies, a group of literatures have reported that stathmin-1, HOTAIR and SUMO1P3 [30]–[32] were correlated with metastasis and prognosis in gastric cancer patients, but it was notable that the testing of these biomarkers were usually high-cost, and conventionally require the fresh gastric cancer tissues. Except these limitations, to the best of our knowledge, the researches of these biomarkers were preclinical, and have not yet been carried out on a large scale in practice. By contrast, D-dimer was a classic marker, which was easy and convenient to test and could be measured in preoperative prediction.

In determining the diagnostic performance of D-dimer levels for peritoneal dissemination, we found that plasma D-dimer was an effective diagnostic marker for peritoneal dissemination in gastric cancer with an AUC of 0.833 (95% CI: 0.780–0.885) and an accuracy rate of 82.59%. According to our findings, plasma D-dimer could be served as a valuable predictor for peritoneal dissemination in gastric cancer. Elevated preoperative D-dimer levels may provide valuable information for determining the treatment and predicting the prognosis of these patients. What's more, our results also indicated that these patients should receive adjuvant therapy even at the earlier stages and are of important to pay more attention during the follow up. But the diagnostic utility of D-dimer levels would need confirmation with clinical studies using large patient populations.

In summary, high levels of D-dimer are associated with peritoneal dissemination and shortened survival time in gastric cancer patients. A plasma D-dimer analysis is an inexpensive, non-invasive and simple method that may be useful as a guide in predicting the dissemination and prognosis of the disease in patients with gastric cancer. This biomarker may also be beneficial in determining the appropriate candidates for adjuvant therapy after surgery and in monitoring cases at high recurrence risk after curative therapies.
